# Providing rapid feedback to residents on their teaching skills: an educational strategy for contemporary trainees

**DOI:** 10.5116/ijme.56dc.908a

**Published:** 2016-03-20

**Authors:** Rachel J. Katz-Sidlow, Tamar G. Baer, Jeffrey C. Gershel

**Affiliations:** 1 Lewis M. Fraad Department of Pediatrics, Jacobi Medical Center, Albert Einstein College of Medicine, USA; 2 Department of Pediatrics, Columbia University Medical Center, USA

**Keywords:** Feedback, graduate medical education, millennials, resident-as-teacher program

## Abstract

**Objectives:**

The objective of this study was to assess the attitudes of contemporary residents toward receiving rapid feedback on their teaching skills from their medical student learners.

**Methods:**

Participants consisted of 20 residents in their second post-graduate training year. These residents facilitated 44 teaching sessions with medical students within our Resident-as-Teacher program. Structured, written feedback from students was returned to the resident within 3 days following each session. Residents completed a short survey about the utility of the feedback, whether they would make a change to future teaching sessions based on the feedback, and what specifically they might change. The survey utilized a 4-point scale (“Not helpful/likely=1” to “Very helpful/likely=4”), and allowed for one free-text response. Free-text responses were hand-coded and underwent qualitative analysis to identify themes.

**Results:**

There were 182 student feedback encounters resulting from 44 teaching sessions. The survey response rate was 73% (32/44). Ninety-four percent of residents rated the rapid feedback as “very helpful,” and 91% would “very likely” make a change to subsequent sessions based on student feedback. Residents’ proposed changes included modifications to session content and/or their personal teaching style.

**Conclusions:**

Residents found that rapid feedback received from medical student learners was highly valuable to them in their roles as teachers. A rapid feedback strategy may facilitate an optimal educational environment for contemporary trainees.

## Introduction

Both the Liaison Committee on Medical Education (LCME) and the Accreditation Council for Graduate Medical Education (ACGME) require that residents receive the proper support to develop as educators.^1^^,^^2^  To fulfil this mandate, Resident-as-Teacher programs are becoming increasingly prevalent in graduate medical education.^3^^-^^5^

As residents put into practice the skills they acquire from Resident-as-Teacher training, feedback provided by the learners may facilitate residents’ development as proficient teachers.^6^^-^^10^ Attention to the timing of such feedback for residents is more important than ever. Today’s residents are members of the “Millennial” generation, defined as those individuals born between the early 1980’s until approximately the year 2000. This population cohort, also sometimes referred to as “Generation Y,” has been raised with technology and its inherent immediacy. Along these lines, Millennials in the business workplace expect and value immediate and ongoing feedback on their work performance.^11^^-^^13^ Similarly, Tuck et al. found that a majority of contemporary residents would like to receive feedback “always or often” on their teaching skills.^10^

Within the clinical education environment, medical students are well-positioned to provide rapid and ongoing feedback to residents. Students spend significant amounts of time learning from residents,^3^^,^^14^-^16^ and are reliable and important judges of effective faculty and resident teaching.^16^^-^^19^ 

In this study, residents received feedback on their teaching skills from their medical student learners. It is important to note that, in general, receptiveness to feedback depends upon “sender credibility,” and is influenced by the recipient’s respect for the individual providing the feedback.^20^^,^^21^ Therefore it is conceivable that residents would discount or devalue feedback from medical student learners. Nevertheless, there is evidence that residents have positive attitudes toward receiving feedback from their junior learners.^9^^,^^10^^,^^15^

What is not yet known is how residents would react to receiving rapid feedback from medical students, and how they might use the rapid feedback to formulate targeted changes to their future teaching. We sought to evaluate Millennial residents’ receptiveness toward a rapid feedback system within our Resident-as-Teacher Program, and to assess whether such feedback could have a direct impact on how residents choose to teach.

## Methods

### Study design and participants

For nearly a decade, our training program has provided a Resident-as-Teacher course spanning all 3 years of residency. The program was developed by the first author on this paper, who serves as departmental Director of Medical Student Education (DMSE). Learning objectives for our residents in their PGY-2 year (i.e. in their second year of post-graduate training) include gaining proficiency in facilitating small-group sessions. To this end, PGY-2 residents are assigned to lead up to 4 sequential, weekly case-based Resident-as-Teacher conferences with third-year medical students. These teaching conferences are scheduled during each resident’s required one month rotation in developmental pediatrics. The number of sessions assigned to each resident varies according to resident availability (e.g. when the resident is not post-call), and how well the dates of the clerkship overlap with the resident’s schedule. Residents are provided clinical cases from the Council on Medical Student Education in Pediatrics curriculum, and are asked to explore up to 4 of these cases with students during an hour-long session. 

We chose this discrete teaching experience as the venue for introducing a “rapid feedback” system for PGY-2 residents on their small-group teaching skills.  The goal was to provide these residents targeted feedback from their medical student learners in time for the residents to make changes, if they chose to do so, by the next week’s session. The rapid feedback system was piloted in August 2013 and then instituted as a standard component of the Resident-as-Teacher program.

After each teaching session, the DMSE routinely emails a brief feedback form to the medical students who have attended. Sessions are held on Friday mornings, and students are asked to send their responses back to the DMSE no later than Sunday evening. Students are informed that their responses will be de-identified and forwarded to the resident in a group format, in order for the resident to be able to enhance his or her teaching for future teaching sessions. Students are asked 4 questions about each session: “What were the greatest strengths of the session? What is one thing that could be done differently to improve the session? What were you confused about during the session? What were two things you learned during the session?” All questions are answered as free-text responses.

The DMSE de-identifies all student responses and emails them in a group format back to the resident within a 3 day time period, i.e. no later than 5 PM on Monday.

The study period was from September 2013 to September 2015. Study participants consisted of a convenience sample of 20 consecutive PGY-2 residents who were assigned in this time period to lead these case-based teaching sessions with medical students as part of our Resident-as-Teacher program.

The project was approved by the Institutional Review Board of the Albert Einstein College of Medicine as an exempt study.

### Data-collection method

Residents in this study were asked to complete a brief survey about the rapid feedback they had received from their medical student learners. Residents were asked 3 questions: “How helpful is it to receive feedback from the students on your sessions? (Not helpful=1, somewhat helpful=2, helpful=3, very helpful=4). How likely are you to make a change in future sessions based on this feedback? (Not likely=1, somewhat likely=2, likely=3, very likely=4). What might you change? (Free text response).” Residents also had the ability to send back additional free-text comments along with their responses to the 3 survey questions, if they desired to do so.

Residents were sent up to 2 reminder emails to complete the survey. Residents were informed via email that their de-identified responses would be analyzed in a research project about the rapid feedback system, and they were given the opportunity to opt out of the study.

### Data analysis

Other than a simple tally of the number of student responses received during the study period, the students’ responses were not reviewed or analyzed for this research project.

Results of the resident survey, including numerical responses and free-text comments were imported into a Microsoft Word table. Free-text responses were hand-coded by the first author on this paper, and underwent qualitative analysis to identify possible themes. Additional resident comments that specifically related to the rapid feedback process were also tabulated. Statements that were favorable toward the rapid feedback intervention were coded as “positive,” while unfavorable comments were considered “negative” statements.

**Table 1:** Residents’ responses to “What might you change?” after receiving feedback from medical student learners on their teaching

Residents’ proposed changes	Selected resident quotes
Theme 1. Session content	
Modify the amount of content taught [Total number of comments /subtheme: 10]	“I am not sure it’s possible to get through all of the questions in the time allotted . . . but I am going to try!”
	“Next time I do the session I will structure it differently . . . Maybe I will spend ten minutes or so [each] on six topics.”
Increase focus on teaching clinical reasoning [Total number of comments / subtheme: 7]	“I will give more concrete approaches on how to differentiate one diagnosis from another.”
	“[I will] add 3 differential diagnoses and point out ways to differentiate between them.”
Highlight the “big picture” [Total number of comments / subtheme: 5]	“I plan to make a slide giving an overview of the cases that will be presented.”
	“I would summarize the main point from the case at the end.”
Increase focus on patient management [Total number of comments / subtheme: 5]	“I will try to focus more on treatment options.”
	“I will have them discuss a more concrete management plan.”
Theme 2. Teaching style	
Provide visual aids [Total number of comments / subtheme:12]	“In the future if there is an anatomy-related scenario, I will [provide] a picture so the students can better visualize.”
	“[I will] use the white board to write down our differential."
Make session more interactive [Total number of comments / subtheme: 6]	“I will take one of the cases and have the students ask me any more information they would like, rather than giving the case to them.”
	“I will now include more interactive cases in the session.”
Include quiet students [Total number of comments / subtheme: 2]	“I will incorporate the suggestion to ask questions in a way that allows those who are quieter to participate.”
	“I plan on incorporating the students’ suggestions, such as calling on students.”
Adjust personal “energy” [Total number of comments / subtheme: 2]	“[I will] be more energetic.”
	“It was helpful to know that I was sometimes jumping in too soon, so will try and sit back and let them discuss more rather than talking so much!”

## Results

Between September 2013 and September 2015, 20 individual PGY-2 residents led a total of 44 teaching sessions to small groups of medical student learners. Each small group session was attended by 2-5 medical student learners, with an average of 4 medical students per session, totaling 182 student feedback encounters. Fifteen residents facilitated between 2-4 sequential weekly sessions, and the remaining 5 residents facilitated one session each. The resident survey response rate was 73% (32/44). Ninety-four percent (30/32) of residents rated the rapid feedback process as “very helpful,” with the remainder rating the intervention as “helpful.” Ninety-one percent (29/32) said they were “very likely” to make a change in subsequent sessions based on the feedback received.  Residents’ free-text answers to the survey question “What might you change?” were coded into two themes: modifications relating to session content, and those that related to residents’ personal teaching styles (Table 1). Residents also submitted a total of 12 additional comments specific to the rapid feedback intervention; 11 comments were positive (Table 2). The 1 comment coded as negative stated “The only drawback of the rapid feedback was that I believe the students lost anonymity, possibly making more critical feedback less likely.” No residents opted out of the study.

**Table 2:** Selected additional comments from residents specific to the rapid feedback intervention

“I am glad for the feedback I received from the prior week because it seems like the new students appreciated those changes that I implemented.”
“The immediate feedback is very useful because I can remember exactly how the session went and what the suggestions are about.”
“It's reassuring that some of the feedback that I implemented in the sessions worked and the students felt they learned the material a lot better.”
“Thanks again for providing this opportunity to develop our teaching skills.”
“It was a great experience working with the students. I had the opportunity to hone my teaching skills, and I really appreciated the rapid feedback.”
“Overall receiving feedback from the students was extremely helpful because it allowed me to tailor the sessions towards what they wanted and in turn they remained interested and enthusiastic during the sessions which made the teaching more fun/interactive.”

## Discussion

Resident-as-Teacher programs commonly teach residents how to provide feedback to junior learners.^4^^,^^8^ Within the context of a Resident-as-Teacher program, residents simultaneously need feedback themselves in order to learn how they are performing as teachers. We sought to assess residents’ attitudes toward receiving rapid feedback about their teaching abilities from medical student learners. In our study, residents received structured written feedback from medical student learners within the 3 days after their teaching sessions. We found that residents appreciated receiving rapid feedback from medical student learners, finding such feedback highly valuable.

Our findings may relate in part to the characteristics of today’s residents, who are members of the Millennial generation. One characteristic of this generation is their desire for and appreciation of timely, ongoing feedback in the workplace.^11^^-^^13^ In addition, Millennials tend to prefer a flat hierarchy in the workplace^22^ and as a group may therefore be particularly comfortable receiving feedback from junior learners. Some suggest that despite their desire for feedback, Millennials may have difficulty hearing negative feedback.^11^^,^^13^ We found, however, that our residents universally rated rapid student feedback on both their teaching strengths and weaknesses as “very helpful” or “helpful.” Over 90% of residents were “very likely” to make a change to their teaching based on medical student feedback; proposed changes related to both session content and personal teaching style.

Limitations to the study include that students were not formally trained to give feedback, and may not know how to do so. However, we found that by providing students with 4 specific questions they were able to identify specific teaching strengths of their residents, as well as areas for improvement. Students were aware that their responses would be transmitted in a group format, and that their comments would be de-identified. As the one resident comment provided in the Results section suggests, it is possible that students were hesitant to provide unfavorable feedback to residents despite knowing that the comments would be de-identified. Prior research has similarly found that residents reported a belief that “student apprehension” was a barrier to feedback.^10^ Nevertheless, in our study, medical student learners routinely provided targeted suggestions as to how residents could improve the sessions, with 91% of residents stating they were “very likely" to make a change to their teaching based on the feedback received. Secondly, this study was performed with Pediatrics residents in a public hospital with a well-established departmental Resident-as-Teacher program; it is possible that our field, or our training program in particular, attracts residents who are particularly receptive to medical student feedback. Finally, this study did not assess whether receiving rapid student feedback and implementing changes would result in improved resident teaching over time. Nevertheless, prior research in other settings has shown that the receipt of rapid feedback, and even the anticipation of receiving rapid feedback, can improve teaching performance.^23^^,^^24^ In addition, there is evidence that residents’ teaching ratings improve after they receive structured written feedback from junior learners.^7^^-^^9^   

## Conclusions

Residents in our study found rapid feedback from medical student learners to be highly valuable, and more than 90% of residents planned to make a specific change to their subsequent teaching sessions based on the feedback received. Proposed changes were specific, and included modifications to session content as well as to their personal teaching style.

We recommend that rapid feedback for residents on their teaching skills be incorporated as an important feature of a Resident-as-Teacher program. A rapid feedback strategy is consonant with Millennial resident preferences and may facilitate an optimal educational environment for this group. 

### Conflict of Interest

The authors declare that they have no conflict of interest.
